# *Mycobacterium avium* Subspecies *paratuberculosis* Drives an Innate Th17-Like T Cell Response Regardless of the Presence of Antigen-Presenting Cells

**DOI:** 10.3389/fvets.2020.00108

**Published:** 2020-03-17

**Authors:** Justin L. DeKuiper, Hannah E. Cooperider, Noah Lubben, Caitlin M. Ancel, Paul M. Coussens

**Affiliations:** Department of Animal Science, Michigan State University, East Lansing, MI, United States

**Keywords:** *paratuberculosis* (MAP), IL-23, IL-17, inflammation, Johne's disease, IL-17 A

## Abstract

The gastrointestinal disease of ruminants is clinically known as Johne's disease (JD) and is caused by *Mycobacterium avium* subspecies *paratuberculosis* (MAP). An accumulative effect by insensitive diagnostic tools, a long subclinical stage of infection, and lack of effective vaccines have made the control of JD difficult. Currently lacking in the model systems of JD are undefined correlates of protection and the sources of inflammation due to JD. As an alternative to commonly studied immune responses, such as the Th1/Th2 paradigm, a non-classical Th17 immune response to MAP has been suggested. Indeed MAP antigens induce mRNAs encoding the Th17-associated cytokines IL-17A, IL-17F, IL-22, IL-23, IL-27, and IFNγ in CD3+ T cell cultures as determined by RT-qPCR. Although not as robust as when cultured with monocyte-derived macrophages (MDMs), MAP is able to stimulate the upregulation of these cytokines from sorted CD3+ T cells in the absence of antigen-presenting cells (APCs). CD4+ and CD8+ T cells are the main contributors of IL-17A and IL-22 in the absence of APCs. However, MAP-stimulated MDMs are the main contributor of IL-23. *In vivo*, JD+ cows have more circulating IL-23 than JD– cows, suggesting that this proinflammatory cytokine may be important in the etiology of JD. Our data in this study continue to suggest that Th17-like cells and associated cytokines may indeed play an important role in the immune responses to MAP infection and the development or control of JD.

## Introduction

*Mycobacterium avium* subspecies *paratuberculosis* (MAP) is the causative agent for the clinical onset of Johne's disease (JD) in ruminants. A MAP infection of the ileum leads to chronic diarrhea and reduces the ability of an animal to absorb nutrients due to inflammation and disruption of the intestinal lining. Clinical JD leads to early culling, reduced milk production, and/or premature death. The cumulative effects of JD are a rising concern to both the animal welfare and the dairy industry. Dairy operations infected with MAP may have risen by ~23% from 2007 to 2013 (68 to 91%) according to the National Animal Health Monitoring System and more recent studies [NAHMS; (1, 2)]. The resulting growth in JD–impacted dairy operations may have concurrently resulted in an increased economic loss to the US dairy industry of $1.3 billion from $200 million (3). The cumulative effects of a long subclinical stage of infection, a lack of an effective vaccine, and insensitive diagnostic tools have made it difficult to control JD. Defining the protective immune responses to MAP has also been difficult. Recent studies suggest that MAP may induce an early or pre-clinical Th17-like immune response (4) in addition to the traditional Th1 and Th2 responses that have been extensively studied in both experimental and natural infections with MAP (5–12).

Th17 cells produce IL-17A, IL-17F, and IL-22 in response to IL-23 acting through the IL-23 receptor (IL-23R) (13) on αβ and γδ T cell surfaces (4, 14, 15). Antagonistic to Th17, the IL-27 and the proinflammatory IFNγ typically are inhibitors of the Th17 pathway (16, 17). IL-27 limits IL-17 responses through the inhibition of RORγ(c) (17) and is a known inhibitor of Th17 in humans and mice (18, 19), while IFNγ inhibits IL-23R expression (16). IL-22 and IL-17F have a synergistic effect with IL-17A (20, 21). Although IL-17A has a much more profound effect on epithelial cells than IL-17F (22), IL-17F is increased in IL-17A-deficient mice and can cause inflammation in the absence of IL-17A (21). The Th17 cells are not limited and produce other Th class cytokines, such as IFNγ and IL-10 (23). The Th17 cells co-expressing IFNγ and IL-17A also express IL-17F (24). Support for the notion that MAP stimulates a Th17 response can be found in studies with other mycobacteria, such as *Mycobacterium tuberculosis* and *Mycobacterium bovis*, which are known to stimulate Th17 cytokines (25, 26). The expression of IL-17A, IL-17F, IL-22, and IL-27 is considered essential in a protective vaccine against *M. bovis* (27). IFNγ was also positively correlated with the expression of IL-17A in *M. bovis* vaccination studies (27). The expression of IL-27, along with IL-17, is also associated with *M. tuberculosis* in humans (28–30).

In current literature discussing MAP infection, the mean relative percent of IL-23R expressing CD4+, CD8+, and TCR1+ cells are increased in MAP-ELISA test-positive cows (JD+) relative to ELISA-negative cows (JD–) (31). Stimulation of peripheral blood mononuclear cells (PBMCs) from healthy JD– cows with MAP increased the mean relative percent of T cell subtypes that expressed IL-23R (31). Furthermore, mRNAs encoding cytokines that direct T cells toward a Th17-like phenotype were upregulated in subclinical JD+ and even JD– PBMCs exposed to MAP in culture (IL-6, IL-1, IL-23, and IL-17A) (31, 32). This is also true in MAP-infected monocyte-derived macrophages (MDMs) (primarily IL-1β, IL6, and IL-23) (33). Naïve helper T cells (CD4+ CD25–) from healthy JD– cows also show increased IL-17A mRNA expression when cocultured with autologous MAP-infected MDMs (32). *In vivo*, IL-17A and IL-6 are upregulated in early-stage MAP-infected lesions (Grade 1) (34), but not in late infection/clinical Johne's disease lesions, suggesting that Th17 responses to MAP are subject to the same T cell exhaustion that occurs with Th1-like cells in late clinical JD (35). The same pattern of IL-17A expression is found in plasma from cows with JD ELISA scores correlating to progressing stages of infection (31). Both IL-23 and IL-17A are specific to a Th17 response, yet neither protein has been conclusively analyzed in bovine antigen-presenting cells and T cells. The intent of this study was to examine Th17 or Th17-like responses to MAP in CD3+ T cells, determine which T cell subtypes are responsible for producing Th17-associated cytokines, and determine if antigen-presenting cells (APCs) are necessary for the T cell expression of Th17 cytokines during the MAP stimulation. Since αβ T cells are MHC-restricted, we included not only MDMs as a potential MHC source but also B cells as another possible source of antigen presentation to T cells as well (36). Thus, we hope to provide new insight into the potential markers for protective immune responses against JD and begin to understand the cause of chronic inflammation in MAP-infected tissues via the IL-23/Th17 inflammatory pathway.

## Materials and Methods

### Study Animals

Up to 10 mature Johne's ELISA test-negative Holstein cows with a similar lactation number (2nd−3rd lactation) were used in this study as previously described in (31) and the sample number (*n*) described in the results. Briefly, JD– cows were sourced from the Michigan State University Dairy Cattle Research and Teaching Center based on an extremely low herd prevalence of MAP infection combined with MAP-negative environmental testing results via fecal sampling from the barn floor for MAP (by fecal PCR) as described previously (37). All protocols for animal handling, use, and sampling were reviewed and approved by the Michigan State University Animal Use and Care Committee.

### Plasma Samples

Plasma used for IL-23 ELISA were obtained from banked plasma stocks stored at −80°C for <5 years. As outlined in prior studies (31), JD ELISA scores were determined at the time of collection following the manufacturer's protocol and the guidelines of commercially available IDEXX ELISA for serum samples from cows. These assays were conducted by AntelBio, Northstar Cooperative Laboratories (Grand Ledge, Michigan). All associated numbers and coordinating JD status ranges are predetermined by the manufacturer of the assay. The diagnostic IDEXX MAP Antibody ELISA used to analyze the plasma samples suggests that a sample with S/P score (referred as OD in this paper) >0.55 is considered as JD–positive, samples scoring under 0.45 OD are considered negative, and OD values in-between are labeled as suspect.

### Preparation of T Cells, B Cells, and Monocyte-Derived Macrophages

Whole blood (30 ml) was collected into 10 ml Vacutainer tubes containing the anticoagulant acid citrate dextrose using 21-gauge (ga) double-sided needles via coccygeal venipuncture and used for all PBMC isolations. A standard Percoll gradient centrifugation protocol (37) was used to isolate PBMCs from whole blood. PBMCs were counted using a Beckman Coulter Counter and plated at a density of 4.2 × 10^6^ in a 96-well flat-bottom culture plate to generate a plating density of about 42K monocyte-derived macrophages (MDMs; estimating 10% of PBMCs). The monocytes were allowed 6 h to adhere to plate surfaces. The cells were then rinsed (4×) with warm PBS to remove non-adherent cells, followed by a 5-day incubation period to allow differentiation into macrophages. On day 5, CD3+ T cells were isolated from fresh PBMCs using a positive selection of the CD3 surface molecule and the magnetic-activated cell sorting (MACS) microbeads (α-ms IgG1; Miltenyi Biotec) as directed by the manufacturer. Briefly, the PBMCs were incubated with an α-bovine CD3 mouse IgG1 antibody (Table 1) at 4°C for 30 min followed by rinses (3×). The cells were then incubated with α-ms IgG microbeads (#130-048-402; Miltenyi Biotec) at 4°C for 30 min followed by rinses (3×), resuspension, and placement in MS columns (#130-042-201; Miltenyi Biotec) on an OctoMACS magnet. Once in the columns, the cells were rinsed (3×) to remove any non-CD3+ cells. The MS columns were then removed from the magnet to allow CD3+ cells to rinse from the column. The cells were then counted again using a Beckman Coulter counter and added to MDMs or cultured alone at densities between 6 and 400 K cells/well depending on the cow. B cell and T cell cocultures were generated from fresh PBMC cultures as described above, with simultaneous incubation of an α-bovine sIgM mouse IgG1 antibody (Table 1) and an α-bovine CD3 antibody separate from the CD3+- only and MDM culture generations. The CD3+ and sIgM+ cells were plated at densities between 11.K and 500K cells/well depending on the cow. Isolated CD4, CD8, and TCR1 T cells (Table 1) were positively selected from fresh PBMCs as described above for CD3+ selection and plated at densities between 51 K and 1.68 ×10^6^ cells/well depending on the cow. The treatment plating densities were equal to the plated control density. All incubations were completed in RPMI 1640 media with 10% fetal bovine serum, 1% penicillin/streptomycin, and 1% Fungizone at 39°C and 5% CO_2_ for 18 h unless otherwise noted.

**Table 1:** Primary and secondary antibody list.

**Antibody**	**Dilution**	**Company**	**Specificity**	**Clone**	**Isotype**
CD4	1:100	WSU	Bovine	IL11A CACT138A	IgG2a IgG1
CD8	1:100	WSU	Bovine	BAQ111A CACT80C	IgM IgG1
TCR1	1:100	WSU	Bovine	GB21A CACTB814	IgG2b IgG1
CD3	1:100	WSU	Bovine	MM1A	IgG1
sIgM	1:100	WSU	Bovine	BIG73A	IgG1
**Fluorophore**	**Dilution**	**Company**	**Specificity**	**Clone**	**Target**
FITC	1:1000	eBioscience	Mouse	m2b-25g4	IgG2b
R-PE	1:200	Invitrogen	Mouse	γ2a	IgG2a
PE-Cy7	1:200	eBioscience	Mouse	m1-14d12	IgM

*WSU, Washington State University*.

### MAP Culture and Treatment

Middlebrook 7H9 media with 10% oleic acid dextrose catalase with 0.2% Mycobactin J supplementation was used to grow isolated MAP cultures (American Type Culture Collection Strain #19698) (32, 38, 39) and maintained at 38°C in log phase until use. Following Janagama et al. (40), the MAP cultures with an OD^600nm^ of 0.6 were removed from culture media by centrifugation and the pellets were resuspended and rinsed (3×) with warm phosphate-buffered saline (PBS) using repeated cycles. To reduce clumping, a 25-ga syringe was used during the resuspension of MAP in PBS. The MAP cell concentrations were estimated as described previously (40), where an OD 600 nm of 0.3 is set approximately equal to 10^9^ bacteria/ml. The potential contamination of MAP cultures was monitored prior to use by inoculation of the brain–heart infusion media with MAP culture aliquots and incubation for 72 h at 37°C.

As a positive control, separate cultures of cells were treated with 25 μg/ml of the general T cell stimulant pokeweed mitogen. The test cultures received a treatment of intact MAP at a multiplicity of infection (MOI) of 2. The CD3+ cultures had an additional test group which received 10 μg/ml of purified protein derivative of Johne's (PPDj). All groups contained a “control” group that is defined as without treatment or stimulation. After incubation, the cultures were pelleted at 600 × g for 5 min at 4°C to aspirate the supernatant. The cells were then rinsed in cold PBS and spun down again. After removing the PBS, the cells were frozen at −80°C until downstream processing for RNA or protein.

A set of isolated CD3+ T cells were cocultured with MDMs. After the treatment, the non-adherent or unbound cells (CD3+) were separated from the MDMs by aspiration and collection of subsequent PBS rinses (3×). EDTA was not used in the PBS as it may have removed the MDMs from the plate surface as well. The CD3 and CD14 primers (Table 2) were used to analyze the purity of the separated cultures by RT-qPCR. The resulting cultures included one containing well-separated CD3+ T (~90%) cells and another with both MDMs and CD3+ T cells which were most likely still bound to the adhered MDM cells.

**Table 2:** Taqman primer list.

**Cytokine**	**Assay ID**	**Entrez gene ID**
IL-22	Bt03261459_m1	507778
IL-17A	Bt03210252_m1	282863
IL-23A	Bt04284624_m1	511022
IL-17F	Bt04309062_m1	506030
IL-27	Bt04298832_m1	614927
IFNg	Bt03212723_m1	281237
CD3d	Bt03225300_m1	281053
CD14	Bt03212325_g1	281048
PPIA	Bt03224615_g1	281418
TBP	Bt03241948_m1	516578

### Cell Surface Staining and Flow Cytometry for Culture Validation

For cell surface staining, the isolated cells were washed in PBS and pelleted at 600 × g for 5 min at 4°C and then resuspended in primary antibody cocktail diluted in sterile First Wash Buffer [1× PBS with 10% acid citrate dextrose, 2% heat-inactivated horse serum (Gibco), and 0.09% sodium azide] (Table 1). The plates were incubated at 4°C for 30 min, washed, and pelleted. The supernatants were aspirated from the wells and the cells were resuspended in secondary antibody cocktail diluted in sterile First Wash Buffer (Table 1). The incubation of cells with secondary antibody for 30 min at 4°C was followed by another wash. The cells were then pelleted, resuspended, and fixed in 1× PBS containing 4% paraformaldehyde for 10 min at 4°C. The fixed cells were washed, pelleted, and resuspended in Second Wash Buffer (90% 1× PBS, 10% acid citrate dextrose, and 0.09% sodium azide) and analyzed by flow cytometry (Accuri C6, Becton Dickinson USA, New Jersey). If immediate analysis was unable to be performed, the plates were briefly stored at 4°C. PBMCs were identified on the basis of forward and side scatter properties with log scaling on both the x-axis and the y-axis. Primary gating regarded all events outside of the PBMC gate as cellular debris. Secondary positive gating strategies calculated the purity of the populations by the mean relative percent (MRP) of cells with positive surface marker staining using FCS Express 4 analytical software. Briefly, the MRP of T cell subtype surface markers within gated cells were analyzed by log side scatter on the y-axis and fluorescence intensity on the x-axis. Unstained (without antibodies) and negative primary control (secondary antibodies only) were also included on each plate to determine final gating. CD3+ and CD4+ isolations demonstrated >90% purity for those cell types. sIgM+ and TCR1+ isolations demonstrated better than 80% purity for those cultures and CD8+ was at about 60% purity (Figure S1).

### RNA Extraction and RT-qPCR

Total RNA was extracted from cultures containing CD3+ using RNeasy Mini Kit (Qiagen) as per the manufacturer's instruction. Due to the lower culture sizes of the T cell isotypes, Arcturus PicoPure RNA isolation kit (Applied Biosystems) was used to isolate total RNA from these samples as per the manufacturer's instruction, with DNA digestion using RNase free DNase set (Qiagen). RNA was quantified using a NanoDrop and samples within the 260/280 range of 1.90–2.20 continued for analysis. The samples outside of the range were excluded. Synthesis of cDNA followed isolation using a high-capacity cDNA Reverse Transcription kit (Applied Biosystems) following the manufacturer's instruction. RT-qPCR was performed in triplicate for all genes. Prior studies in our lab demonstrated that Peptidylprolyl isomerase A (PPIA) and TATA-box binding protein (TBP) were ideal housekeeping candidates for our genes (32). PPIA was used as a housekeeping gene for IL-17A, IL-22, and IL-23 (Table 2). TBP was later used as a housekeeping gene for IL-17F, IL-27, and IFNγ. Taqman reagents were used for all RT-qPCR experiments (Table 2) using an Applied Biosystems 7500 qPCR instrument and 7500 software v2.0.6. Ct values were obtained using the software. ΔCt was calculated by subtracting the housekeeping Ct value first from the Ct value of the autologous gene of interest. ΔΔCt was then calculated by the difference of the treatment ΔCt and the control (unstimulated) ΔCt. An analysis compared the control and treatment 2^−ΔΔCt^ values.

### ELISA

The plasma samples were diluted 1:2 and analyzed for circulating IL-23 using pre-made plates and solutions (MyBioSource) following the manufacturer's recommended protocol. All ELISA analytics were performed at 450 nm using Molecular Devices Spectra Max M5 and SoftMax Pro 6.5.1 analytical software.

### Statistics

The IL-23 plasma cytokine levels and cultures with CD3+ T cells were analyzed using Kruskal–Wallis and Dunn's multiple-comparison tests. Isolated T cell subtype culture medians were compared using Wilcoxon–Mann–Whitney tests. Statistical significance was set at *p* ≤ 0.05 (α = 0.05; chance of making a type I error). All statistics were computed using Prism GraphPad statistical analysis software. The samples were subject to Grubb's outlier test to find the extreme outliers and it is mentioned in the text if any extreme outliers were excluded in the results.

## Results

### CD3+ T Cells Cocultured With Monocyte-Derived Macrophages

**Figure 1:**
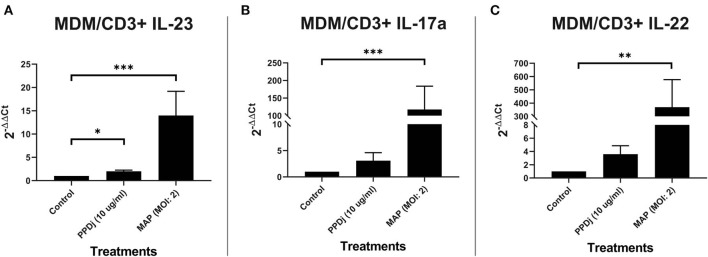
Relative abundance of IL-23, IL-17a, and IL-22 mRNA of cultures containing MDMs cocultured with autologous CD3+ T cells and stimulated with MAP. CD3+ T cells were cultured with 5-day-old MDMs and stimulated with PPDj or MAP (MOI of 2) or left unstimulated for 18 h. Subsequent RNA extraction and qPCR results are shown. The MAP- and the PPDj-stimulated cultures showed a significant upregulation of **(A)** IL-23. Only the MAP-stimulated cultures showed a significant upregulation of **(B)** IL-17A and **(C)** IL-22 as well. Analysis by Kruskal–Wallis and Dunn's multiple-comparison tests. *n* = 6–8/group. *p < 0.05, **p < 0.01, ***p < 0.001.

To further understand the relationship between MDMs plus CD3+ T cells and IL-23, IL-17A, and IL-22 in regard to a MAP-stimulated Th17 response, 5-day old MDMs were cocultured with autologous MAC sorted CD3+ T cells and stimulated with MAP antigen for 18 h. RNA was extracted from each culture, pooled, and analyzed. MAP stimulation (*n* = 8) significantly increased the mRNA encoding the Th17-promoting cytokine IL-23 when compared to the unstimulated control cultures (*n* = 8; *p* < 0.001; Figure 1A). PPDj (*n* = 8) was able to significantly upregulate IL-23 mRNA when compared to the unstimulated control cultures (*p* < 0.05), but to a lesser extent than the live MAP (Figure 1B). Th17-specific mRNA encoding cytokine IL-17A and the Th17-associated cytokine IL-22 were also significantly upregulated when compared to the control cultures (*n* = 7 and *n* = 6, respectively). IL-17A mRNA increased 118-fold in MAP-treated cultures relative to the control untreated cultures (Figure 1B), and IL-22 increased 369-fold in MAP-treated cultures relative to the control untreated cultures (Figure 1C) (*n* = 7 and *n* = 6; *p* < 0.001 and 0.01, respectively). PPDj also tended to increase IL-17A and IL-22 mRNA levels (3.1 and 3.6-fold, respectively) in cocultures relative to untreated cells; however, these differences were not statistically significant in this test. All analyses were completed using Kruskal–Wallis and Dunn's multiple-comparison tests. One extreme outlier was identified for IL-23 within the PPDj group by Grubb's outlier test and is not included in this analysis. A smaller set of MDM plus CD3+ T cell cocultures were rinsed to remove any unbound CD3+ T cells before RNA isolation. When compared to their respective unstimulated controls, the MAP-stimulated MDM cultures with remaining bound CD3+ T cells demonstrated a mean 440-fold increase of IL-17A mRNA relative to the untreated cells (*p* < 0.05; *n* = 3; Figure S2A). The separated MAP-stimulated CD3+ T cells contained 29-fold more IL-17A mRNA than the untreated CD3+ T cells (*p* < 0.05; *n* = 3; Figure S2B). These increased levels of IL-17A were similar to the MAP-treated CD3+-only cultures as shown in Figure 3A. IL-23 mRNA was significantly upregulated in the MAP-stimulated cultures containing MDMs by a mean of 22-fold relative to the untreated cultures (*p* < 0.05; *n* = 3; Figure S2C). However, the separated MAP-stimulated CD3+ T cells exhibited only a median 2.2-fold (*p* = 0.06; *n* = 4; Figure S2D) increase in IL-23 mRNA relative to the untreated CD3+ T cells. IL-22 mRNA did not increase in the MDMs with bound CD3+ coculture (*p* = 0.07; *n* = 4; Figure S2E). However, a 104-fold increase in IL-22 mRNA was observed in the separated MAP-stimulated CD3+ cultures (*p* < 0.05; *n* = 4; Figure S2F). PPDj did not have as profound of an effect on IL-23 mRNA production as it did with IL-17A and IL-22 (*p* ≤ 0.14; Figures S2A–F).

### CD3+ T Cells Cocultured With sIgM+ B Cells

**Figure 2:**
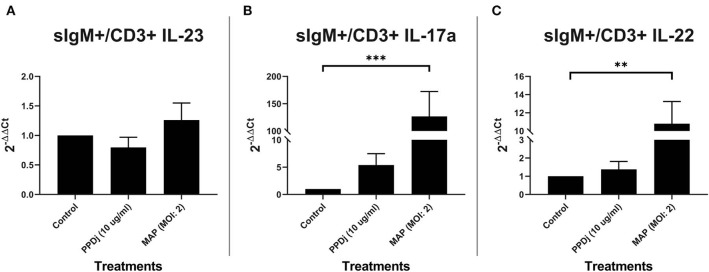
Relative abundance of IL-23, IL-17a, and IL-22 mRNA of cultures containing sIgM+ B cells cocultured with autologous CD3+ T cells and stimulated with MAP. CD3+ T cells were cultured with sIgM+ B cells and stimulated with PPDj or MAP (MOI of 2) or left unstimulated for 18 h. Subsequent RNA extraction and qPCR results are shown. The MAP- and the PPDj-stimulated cultures did not show an upregulation of **(A)** IL-23. Only the MAP stimulation showed a significant upregulation of IL-17A **(B)** and IL-22 **(C)**. Analysis by Kruskal–Wallis and Dunn's multiple-comparison tests. *n* = 6–7/group. *p < 0.05, **p < 0.01, ***p < 0.001.

B cells have the capacity to be antigen-presenting cells (36). In lieu of this characteristic, autologous sIgM B cells and CD3+ T cells were MAC sorted, cocultured, and stimulated with MAP antigen for 18 h as before; the control cultures were not treated with MAP. Unlike the cultures with MDMs, mRNA-encoding IL-23 was not upregulated in MAP- (*n* = 7) or PPDj- (*n* = 6) stimulated cultures when compared to their respective untreated control cultures (*n* = 7; Figure 2A). However, like the MDM coculture, IL-17A mRNA was increased 127-fold in MAP-stimulated cultures when compared to the untreated control cultures (*n* = 7; *p* < 0.001; Figure 2B). Similarly, mRNA-encoding IL-22 was upregulated 11-fold in MAP-stimulated cultures compared to the untreated controls (*n* = 7; *p* < 0.01; Figure 2C). The increase in IL-22 mRNA was significant but numerically much less than that observed with MAP-stimulated MDM cocultures. Again, PPDj slightly increased IL-17A (*n* = 6) and IL-22 (*n* = 6) mRNA, but these differences were not significant (5.4 and 1.4-fold, respectively). One extreme outlier was identified in all PPDj-stimulated groups by Grubb's outlier test, and these values are not included in this analysis.

#### CD3+ T Cell Culture

**Figure 3:**
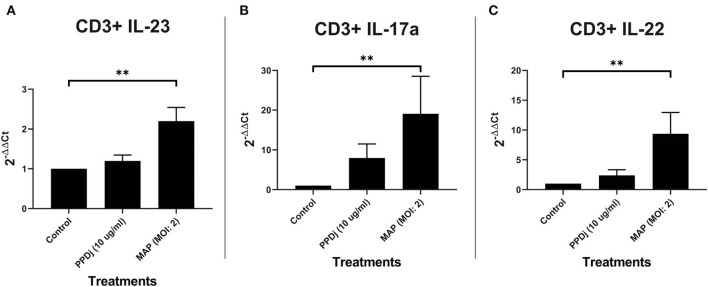
Relative abundance of IL-23, IL-17a, and IL-22 mRNA of cultures containing CD3+ T cells and stimulated with MAP. CD3+ T cells were cultured independently and stimulated with PPDj or MAP (MOI of 2) or left unstimulated for 18 h. Subsequent RNA extraction and qPCR results are shown. The MAP-stimulated cultures showed a significant upregulation of **(A)** IL-23, **(B)** IL-17A, and **(C)** IL-22. Analysis by Kruskal–Wallis and Dunn's multiple-comparison tests. *n* = 8/group. *p < 0.05, **p < 0.01, ***p < 0.001.

Because γδ T cells can be induced to express IL-17A without the aid of traditional APCs (41) and the possibility that MAP stimulation of αβ T cells via TCR-independent mechanisms could also trigger IL-17A production, we next examined CD3+ MAC-sorted T cells stimulated with MAP for 18 h in the absence of any appreciable B cells or monocytes. When cultured without the presence of traditional APCs, CD3+ T cells respond to MAP in a Th17-like manner. IL-23 mRNA is significantly increased by 2.2-fold in CD3+ T cell cultures treated with MAP (*n* = 8) when compared to the control cultures (*n* = 8; *p* < 0.01; Figure 3A). Th17-specific mRNA-encoding cytokines IL-17A (*n* = 8; Figure 3B) and IL-22 (*n* = 8; Figure 3C) are upregulated in MAP-stimulated CD3+ cultures when compared to the unstimulated cultures (*p* < 0.01). When compared to MAP-stimulated MDM plus CD3+ T cell and sIgM+ B cell plus CD3+ T cell cocultures, the average fold change of IL-17A mRNA in MAP-stimulated CD3+-only cultures relative to that of the controls was lower (*p* = 0.13 and 0.15, respectively; Figure S3A). Likewise, the mean fold change for IL-22 mRNA was significantly lower in MAP-stimulated CD3+-only cultures compared to that observed in MDM plus CD3+ T cell cocultures (*p* < 0.05) but was similar to the IL-22 mRNA levels seen in sIgM+ B cell plus CD3+ cell cocultures (Figure S3B). The mean IL-23 mRNA fold change was significantly lower when compared to the 14-fold increase seen with MAP-stimulated MDM plus CD3+ T cell cocultures (*p* = 0.05; Figure S3C). However, this was not significantly different when compared to the 1.3-fold change in sIgM+ B cell and CD3+ T cell cocultures (Figure 2A). One extreme outlier was identified in all MAP-stimulated groups and the PPDj group of IL-23 by Grubb's outlier test and is therefore not included in this analysis.

## αβ T Cells Are the Main Producers of IL-17A and IL-22 in the Absence of Antigen-Presenting Cells

**Figure 4:**
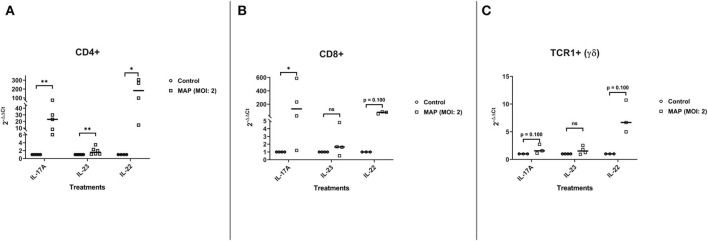
Relative abundance of Th17 mRNA encoding IL-17A, IL-22, and IL-23 from CD4+, CD8+, and TCR1+ (γδ) cell cultures stimulated with MAP or left unstimulated. The isolated CD4+, CD8+, and TCR1+ T cell cultures were stimulated with MAP or left unstimulated for 18 h. Subsequent RNA extraction and qPCR results are shown. **(A)** The CD4+ T cells demonstrated a significant upregulation of all three cytokines (IL-17A, IL-23, and IL-22; *n* = 4–6/group). **(B)** The CD8+ T cells demonstrated a significant upregulation of IL-17A and near significant for IL-22. However, IL-23 was not (*n* = 3–4/group). **(C)** The γδ T cells did not demonstrate a significant upregulation of any of the cytokines (*n* = 3–4/group). Analysis by Wilcoxon–Mann–Whitney tests. One extreme outlier was found by Grubb's test in the “γδ IL-23” group and is not included in the analysis. *p < 0.05, **p < 0.01, ***p < 0.001.

Although γδ T cells are able to produce IL-17A in the absence of APCs (41), CD4+ and CD8+ T cells appear to be the main producers of IL-17A in response to MAP without the aid of traditional APCs (Figures 4A,B). MAP-stimulated CD4+ T cells (*n* = 5) increased the expression of mRNA-encoding IL-17A with a median 23-fold increase compared to the unstimulated CD4+ T cells (*n* = 5; *p* < 0.01; Figure 4A). The MAP-stimulated CD8+ T cells (*n* = 4) upregulated IL-17A mRNA with a median 130-fold increase compared to the non-stimulated cells (*n* = 4; *p* < 0.05; Figure 4B). IL-22 mRNA was also significantly increased (182-fold) in MAP-stimulated CD4+ T cells (*n* = 4) compared to the non-stimulated cells (*n* = 4; *p* < 0.05; Figure 4A), while MAP-stimulated CD8+ cells (*n* = 3) tended to express more (median 79-fold) IL-22 mRNA relative to the unstimulated CD8+ cells (*p* = 0.10; Figure 4B). Rather surprisingly, the γδ T cells (TCR1+) did not significantly increase IL-17A mRNA (*n* = 3; median 1.6-fold) or IL-22 mRNA (*n* = 3; median 6.7-fold; Figure 4C) in response to MAP stimulation relative to the untreated control γδ T cells. IL-23 mRNA was significantly increased in CD4+ T cells (*n* = 6; median 1.6-fold), but not in CD8+ T cells (*n* = 4; median 1.6-fold) or γδ T cells (*n* = 4; median 1.5-fold) compared to the respective controls. Grubb's test identified one extreme outlier in the γδ T cell IL-23 assays that was not included in the results.

## CD3+ T Cells Also Express Enhanced Levels of IFNγ mRNA, but Not at Levels Observed for IL-17A or IL-17F mRNAs

**Figure 5:**
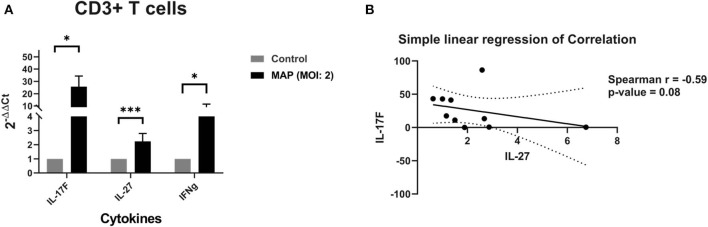
**(A)** Relative abundance of mRNA encoding IL-17F (Th17), IL-27 (anti-Th17), and IFNγ from CD3+ cell cultures stimulated with MAP or left unstimulated. The isolated CD3+ T cell cultures were stimulated with MAP or left unstimulated for 18 h. Subsequent RNA extraction and qPCR results are shown. The CD3+ T cells demonstrated a significant upregulation of mRNA for Th17 cytokine IL-17F, Th1 cytokine IFNγ, and Th17 inhibitory cytokine IL-27 by Wilcoxon–Mann–Whitney tests. *n* = 10/group. *p < 0.05, **p < 0.01, ***p < 0.001. **(B)** Correlation between the quantities of mRNA encoding IL-17F and IL-27 from CD3+ cell cultures stimulated with MAP or left unstimulated. The isolated CD3+ T cell cultures were stimulated with MAP or left unstimulated for 18 h. Subsequent RNA extraction and qPCR results are shown. IL-17F may share a moderate negative correlation with IL-27 by Spearman correlation test. *n* = 10, *r* = −0.59, *p* = 0.081.

MAP-stimulated CD3+ T cells also upregulate the Th17-specific mRNA-encoding cytokine IL-17F by a mean 26-fold when compared to the unstimulated samples (*p* = 0.02; *n* = 10; Figure 5A). The MAP-stimulated CD3+ T cells also upregulate the mRNA-encoding IL-27 slightly by a mean 2.24-fold (*p* = 0.0006; *n* = 10; Figure 5A) and IFNγ at a median 7.7-fold (*p* = 0.0135; *n* = 10; Figure 5A). IL-27 and IL-17F share a moderate negative correlation (Spearman *r* = −0.59), but this is only nearly significant with the sample size we currently have (*n* = 10; *p* = 0.081; Figure 5B).

## Increased IL-23 Levels in JD ELISA Test-Positive Cows

**Figure 6:**
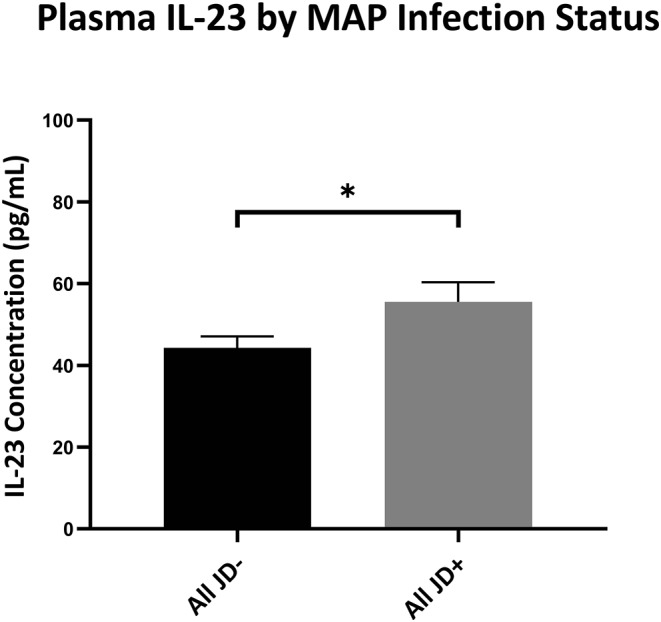
Plasma IL-23 levels of cows based on infection status by IDEXX Johne's ELISA. IL-23 concentrations (pg/ml) circulating in the plasma from the periphery of JD– and JD+ cows as tested by ELISA. Overall JD– cows (*n* = 46) and JD+ cows (*n* = 30). *p < 0.05. Error bars = SEM. Cow *n* is based on the available stocked plasma samples. Unpaired *t*-test was used between overall JD– and JD+ groups.

To help better understand IL-23's importance on the prevalence of IL-17A as previously reported in (31), we looked into the plasma IL-23 levels as it corresponds to the infection status. Indeed JD+ cows had significantly more IL-23 in circulation than the JD– cows had (unpaired Studen's *t*-test; *p* < 0.05) (Figure 6). There were no significant differences in the circulating IL-23 levels in JD+ cows separated in groups according to increasing JD ELISA test score OD values (Figure S4).

## Discussion

Th17 cells and cytokines are likely playing a major role in MAP infection and Johne's disease (31). While it is not yet clear what precise roles Th17 responses and their associated cytokines might have in the progression of Johne's disease, the data presented herein and elsewhere (4, 31–34) suggest that Th17 responses are an important feature of immune cell reactions to MAP. IL-17A is a protective cytokine in some inflammatory diseases (42, 43) and in mycobacterial infections following an early infection (25, 44, 45). Chronic inflammation, a distinct feature of MAP infection and Johne's disease, could be induced by either IL-17A (21) or IL-23 (46, 47). However, IL-17A may play more of a protective role as it seems to be upregulated in early infection but is downregulated in late clinical disease, similar to what is observed with IFNγ (31, 32, 34). Evidence suggests that these patterns of expression may be due to T cell exhaustion in clinical Johne's disease (11, 35). The continuous production of IL-23 from, for example, MAP-infected macrophages could cause epithelial dysregulation and inflammation, which are hallmarks of clinical Johne's disease (46, 47).

MAP is known to upregulate IL-6, IL-23, IL-1β, and TGF-β mRNA expression as early as 1 h post-infection in MAP-infected MDMs (33), indicating that the development of Th17 cells may be promoted by the local cytokine environment near sites of MAP infection. MAP infection appears to increase the mean relative percent of T cells expressing IL-23R (31). Indeed the CD3+ T cells dramatically increased the mRNA production for Th17 cytokines such as IL-17A, IL-22, and IL-23 in the presence of MAP and MDMs. It is likely that a majority of the IL-23 mRNA expression that we observed is from MAP-stimulated or infected MDMs and not T cells, given that the IL-23 mRNA levels from CD3+ T cell-only cultures and cocultures containing CD3+ T cells and B cells are not significant or only slightly increased relative to the control untreated cells. We did observe a slight increase in IL-23 mRNA in CD3+-only cultures, likely due to the known properties of Th1 and Th2 cells that can express IL-23 mRNA (48).

In experimentally and naturally infected JD+ cows, the B cell counts are increased, including activated and memory B cells (37, 49). B cells are also able to produce IL-23 through BCR signaling (50). However, the increase of IL-17A mRNA in sIgM+ B cell plus CD3+ T cell cocultures did not seem to be coincident with the elevated IL-23 mRNA levels as it was in MDM plus CD3+ T cell cultures. There was little or no increase in IL-23 or IL-22 mRNAs in cocultures containing CD3+ T cells and B cells. Our results do not rule out a potential role for IL-17A produced by either B cells or by γδ T cells during early responses to MAP infection. B cells can produce IL-17A independently of IL-23 (51) and this has importance in response to other pathogens (51). Similarly, prior research has shown that γδ T cells also respond during early infection with IL-17A production after direct stimulation of their TCR and without restriction of MHC activation (41). This “innate” IL-17A production may be of limited quantity and/or duration (52). While γδ T cells do not require IL-23 for initial production of IL-17A (43), continued IL-17A secretion may require IL-23 (52).

In the current study, we cultured CD3+ T cells without appreciable traditional APCs present. Indeed CD3+ T cells were able to increase IL-17A (19-fold), IL-22 (9.4-fold), and IL-23 (2.2-fold) mRNA levels in the absence of traditional APCs. Macrophages are a major supplier of IL-23 (33). As seen in Baldwin et al. (52), the production quantity of IL-17A and IL-22 is reliant on the presence of APCs or the quantity of IL-23. The loss of macrophages in our CD3+ cultures resulted in a reduction of IL-23 mRNA and, consequently, a reduction in IL-17A mRNA and IL-22 mRNA. This is largely noticeable in cultures containing MDMs compared to cultures containing only CD3+ T cells. Additionally, cultures containing B cells were unable to upregulate IL-23 mRNA in comparison to CD3+ T cell cultures and thus unable to increase IL-22 mRNA either. IL-17A was unaffected; however, this may likely be due to B cell's ability to secrete IL-17A through pathogen stimulation (51) rather than IL-23 stimulating IL-23R on the T cell surface.

We sought to determine which CD3+T cell subtypes were responsible for producing IL-17A, IL-22, and perhaps IL-23. To this end, we sorted T cell subgroups representing CD4+, CD8+, and TCR1+ (γδ) T cells. These sorted T cell subtypes were cultured with MAP, but without added MDM cells, and analyzed to determine which subtypes were contributing to the production of Th17 cytokines during MAP stimulation. We found that αβ T cells were the main producers of IL-17A and IL-22 mRNAs, with CD4+ cells producing only small amounts of IL-23 mRNA. The CD8+ cells exhibited a larger increase in IL-17A mRNA (129-fold) than either CD4+ cells (23-fold) or γδ T cells (1.55-fold) relative to the unstimulated cells. However, CD4+ cells produced more IL-22 mRNA in response to MAP (182-fold) than either CD8+ (79-fold) or γδ T cells (6.67-fold) relative to the unstimulated cells. These results clearly indicate that T cells can directly respond to MAP with production of Th17-associated cytokine mRNA without significant APCs. Our study is not the first to show the CD4+ cells expressing a Th17 phenotype without the direction or activation of APCs (53). Other studies have indicated that naïve αβ T cells can produce IL-17A and IL-22 without IL-23 as part of an innate response (53). In another study, αβ T cells produced IL-23 mRNA with direct stimulation (48). While we expected to observe significant IL-17A mRNA production by isolated γδ T cells, this was not the case. By isolating γδ T cells from other T cell subtypes, we may have hindered their innate potential to respond to MAP in a Th17 manner as seen in other studies (54). An alternative explanation is that γδ T cells tend to produce significant amounts of both IFNγ and IL-27, which are inhibitors of IL-17A production.

Cells and tissues infected with *M. tuberculosis* or *M. bovis* upregulate IL-17A, IL-22, and IL-23 expression. These infected tissues also exhibit increased levels of IL-17F as well as the Th17 inhibitors IL-27 and IFNγ (27–30). MAP-stimulated CD3+ T cells seem to follow this same pattern of expression. The CD3+ T cells respond to MAP stimulation with increased production of both IL-17A and IFNγ mRNA (mean 11-fold relative to the unstimulated cells). Our data does not allow a distinction between non-classical Th17 cells that also express IFNγ and traditional Th1-like T cells.

Contrary to the decreased IL-17A levels in the periphery of cows with high JD+ ELISA scores (31), IL-23 is still elevated in the plasma of JD+ cows. IL-23 is also a proinflammatory cytokine that can cause inflammation and dysregulation (42, 55, 56). Overall, JD+ cows have more IL-23 in the periphery than the JD– cows have. Our results also suggest that macrophages exposed to MAP are likely a significant source of IL-23. It is well-known that, as JD progresses, more macrophages migrate to the tissue and are infected with MAP (34). If these macrophages are stimulated to produce IL-23 mRNA and protein, as seen in this study and others (33), the continued expression of IL-23 seen here could be a cause for the advanced inflammation (55, 56) and depletion of T cells observed in tissues of advanced JD (34, 56).

MAP induces CD3+ T cells to adopt a Th17-like phenotype even in the absence of APCs as evidenced by the enhanced production of IL-17A, IL-17F, IL-22, and IL-23. However, the continued or robust expression of Th17 cytokines IL-17A and IL-22 during early infection is reliant on the presence of IL-23, which could be supplied by MAP-infected macrophages. MAP-stimulated expression of cytokine mRNA follows similar patterns seen by other mycobacterial infections, including significant increases in IL-22 mRNA and increased expression of mRNA-encoding IFNγ and IL-27 (27–30). sIgM+ B cells are also a likely source of innate MAP-stimulated IL-17A, although further exploration is warranted. Additionally, MAP studies focusing on IL-27 should also be considered. *M. tuberculosis* studies demonstrated an increased protective role by IL-17A, as well as an increased percentage of CCR6+/CD4+ T cells and IL-17+/CD4+ T cells in mice that were IL-27 receptor (R) deficient (57). Neutralizing IL-27 also induced lysosomal acidification in macrophages infected with *M. tuberculosis* and increased mycobacterial killing (58, 59). IL-27 was a source of immune suppression in *Mycobacterium leprae* infection (60). It is possible that the inflammation observed in JD may, at least in part, be attributed to Th17-related cytokines, specifically IL-23. Future studies concerning JD should be directed into a further exploration of these cytokines.

## Data Availability Statement

The datasets generated for this study are available on request to the corresponding author.

## Ethics Statement

The animal study was reviewed and approved by Michigan State University Animal Use and Care Committee.

## Author Contributions

JD helped plan all experiments and performed all cell sorting and isolation. JD also performed all data analysis and supervised HC, CA, and NL. HC, CA, and NL performed Q-RT-PCR studies, assisted in blood collection and processing, assisted in cell sorting, and helped with cell cultures. PC was the overall project supervisor, reviewed all data, worked with JD to plan all experiments and assisted in preparation of the manuscript.

### Conflict of Interest

The authors declare that the research was conducted in the absence of any commercial or financial relationships that could be construed as a potential conflict of interest.

## Supplementary Material

Figure S1 Flow cytometry gating strategy for MACS validation. Strategy includes no stain control (not shown) and negative primary control (secondary only). All events outside of the isolated cell gate are considered debris. CD3+ and CD4+ isolations demonstrated a >90% purity for those cell types. sIgM+ and TCR1+ isolations demonstrated better than 80% purity for those cultures and CD8+ was >60% purity.

Figure S2 Relative abundance of IL-17A, IL-23, and IL-22 mRNA of MDMs and bound CD3+ T cells or CD3+ only after coculture, stimulation with MAP, and unbound cells were rinsed away. CD3+ T cells were cultured with 5-day old MDMs and stimulated with PPDj, MAP (MOI of 2), or left unstimulated for 18 h. Unbound CD3+ T cells were rinsed and analyzed separately. Subsequent RNA extraction and qPCR results are shown. **(A)** MAP stimulated cultures showed a significant upregulation of IL-17A in both **(A)** MDMs with bound CD3+ as well as the **(B)** unbound CD3+ cells (*n* = 3 and 4/group, respectively). MAP stimulated cultures showed a significant upregulation of IL-23 in **(C)** MDMs with bound CD3+ while only a near significant upregulation in **(D)** unbound CD3+ cells (*n* = 3 and 4/group, respectively). MAP stimulated cultures showed a near significant upregulation of IL-22 in **(E)** MDM with bound CD3+ and a significant increase in **(F)** unbound CD3+ cells (*n* = 3 and 4/group, respectively). Analysis by Kruskal–Wallis and Dunn's multiple comparison tests. ^*^*p* < 0.05. ^**^*p* < 0.01. ^***^*p* < 0.001.

Figure S3 Relative abundance of IL-17A, IL-22, and IL23 mRNA of CD3+ cells, MDM/CD3+, and sIgM+/CD3+ cultures stimulated with MAP. CD3+ T cell cultures with and without APCs were stimulated with MAP for 18 h. Subsequent RNA extraction and qPCR results are shown. **(A)** APC containing cultures demonstrated the most upregulation of IL-17A (*n* = 7–8/group). **(B)** MDM containing cultures demonstrated the most upregulation of **(B)** IL-22 (*n* = 7–8/group) and **(C)** IL-23 (*n* = 6–8/group). Analysis by Kruskal–Wallis and Dunn's multiple comparison tests. ^*^*p* < 0.05. ^**^*p* < 0.01. ^***^*p* < 0.001.

Figure S4 Plasma IL-23 levels of cows based on IDEXX Johne's ELISA score. IL-23 concentrations (pg/mL) circulating in the plasma from the periphery of by ELISA. Low JD– (x <0.2; *n* = 29). Mid JD– (0.2 < x0.3; *n* = 9). High JD– (0.3 < x <0.55; *n* = 8). Low JD+ (0.55 < x <1.0; *n* = 6). Mid JD+ (1.0 < x <2.0; *n* = 9). High JD+ (x > 2.0; *n* = 15). Brown-Forsythe ANOVA test and Dunnett's T3 multiple comparisons test were used in the observation of score groups. ^*^*p* < 0.05. Error bars = SEM. Cow *n* is based on available stocked plasma samples.
