# Supplementary figures and images for: Mycobacterium avium Subspecies paratuberculosis Drives an Innate Th17-Like T Cell Response Regardless of the Presence of Antigen-Presenting Cells
Source: Front Vet Sci. 2020 Mar 17;7:108. doi: 10.3389/fvets.2020.00108 (PMC7089878; doi:10.3389/fvets.2020.00108)

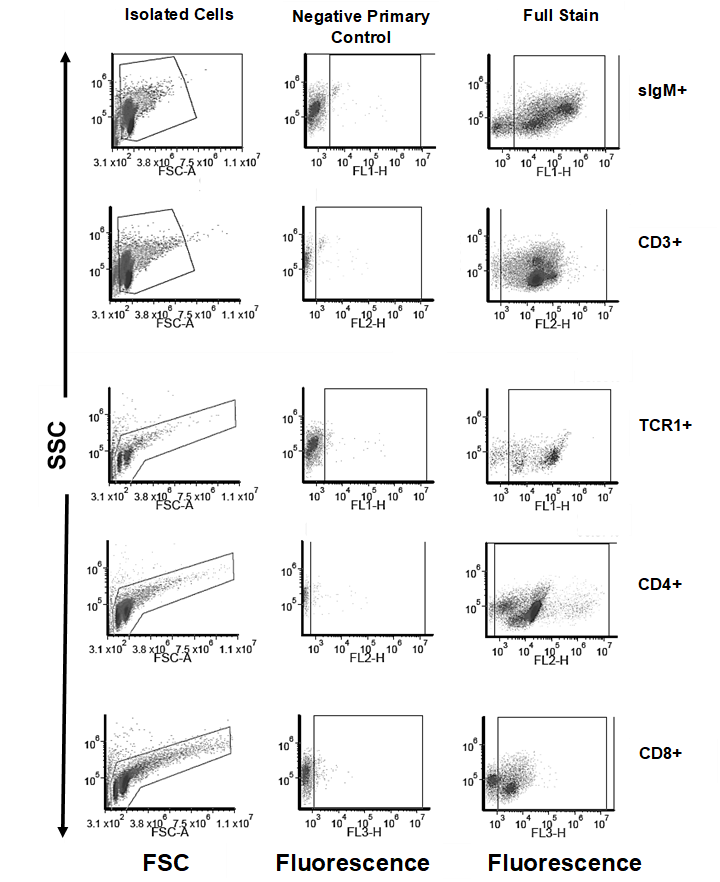

Supplement: Figure S1 — Flow cytometry gating strategy for MACS validation. Strategy includes no stain control (not shown) and negative primary control (secondary only). All events outside of the isolated cell gate are considered debris. CD3+ and CD4+ isolations demonstrated a >90% purity for those cell types. sIgM+ and TCR1+ isolations demonstrated better than 80% purity for those cultures and CD8+ was >60% purity. [file Image_1.TIF]

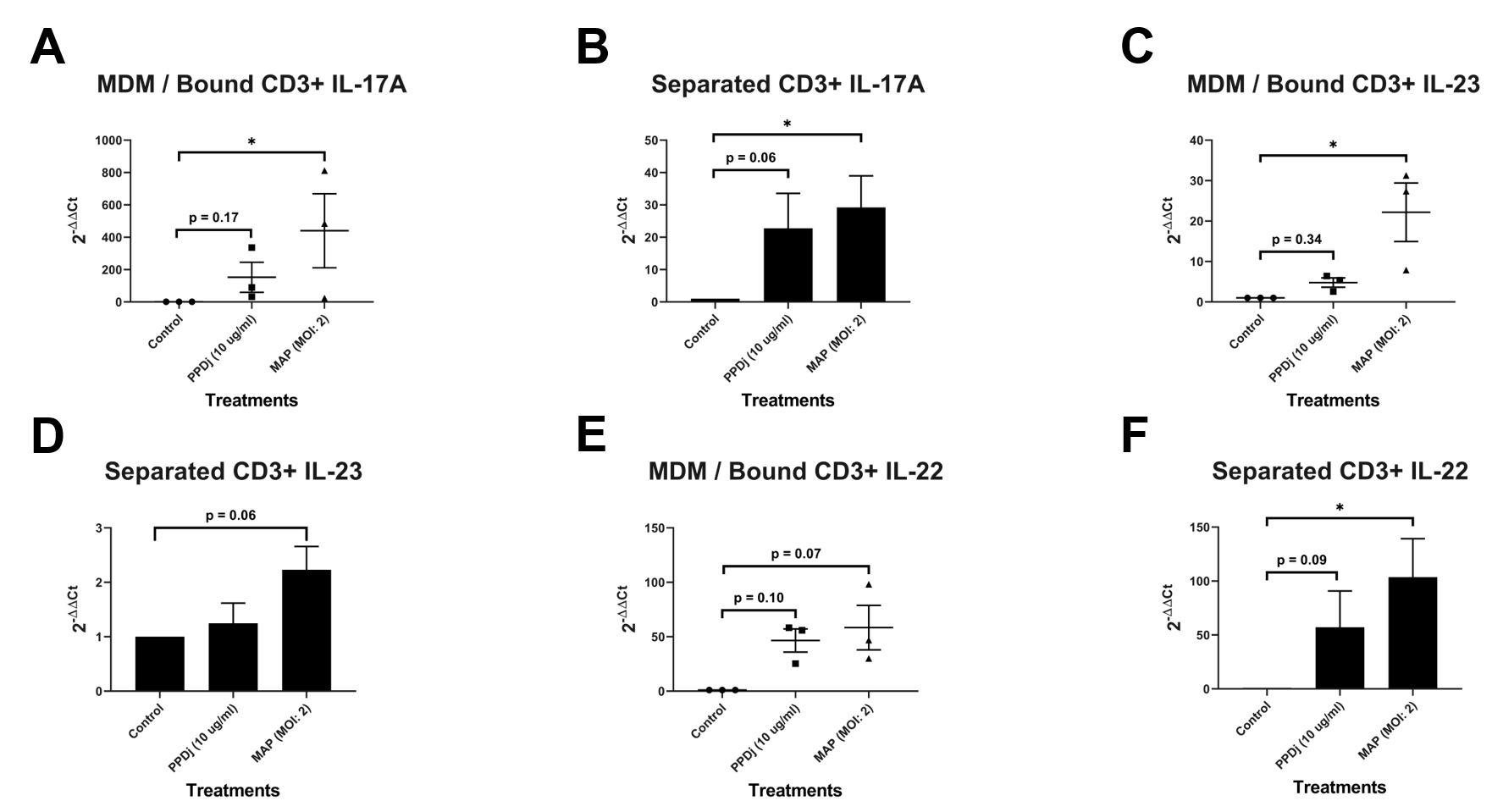

Supplement: Figure S2 — Relative abundance of IL-17A, IL-23, and IL-22 mRNA of MDMs and bound CD3+ T cells or CD3+ only after coculture, stimulation with MAP, and unbound cells were rinsed away. CD3+ T cells were cultured with 5-day old MDMs and stimulated with PPDj, MAP (MOI of 2), or left unstimulated for 18 h. Unbound CD3+ T cells were rinsed and analyzed separately. Subsequent RNA extraction and qPCR results are shown. (A) MAP stimulated cultures showed a significant upregulation of IL-17A in both (A) MDMs with bound CD3+ as well as the (B) unbound CD3+ cells (n = 3 and 4/group, respectively). MAP stimulated cultures showed a significant upregulation of IL-23 in (C) MDMs with bound CD3+ while only a near significant upregulation in (D) unbound CD3+ cells (n = 3 and 4/group, respectively). MAP stimulated cultures showed a near significant upregulation of IL-22 in (E) MDM with bound CD3+ and a significant increase in (F) unbound CD3+ cells (n = 3 and 4/group, respectively). Analysis by Kruskal–Wallis and Dunn's multiple comparison tests. *p < 0.05. **p < 0.01. ***p < 0.001. [file Image_2.TIF]

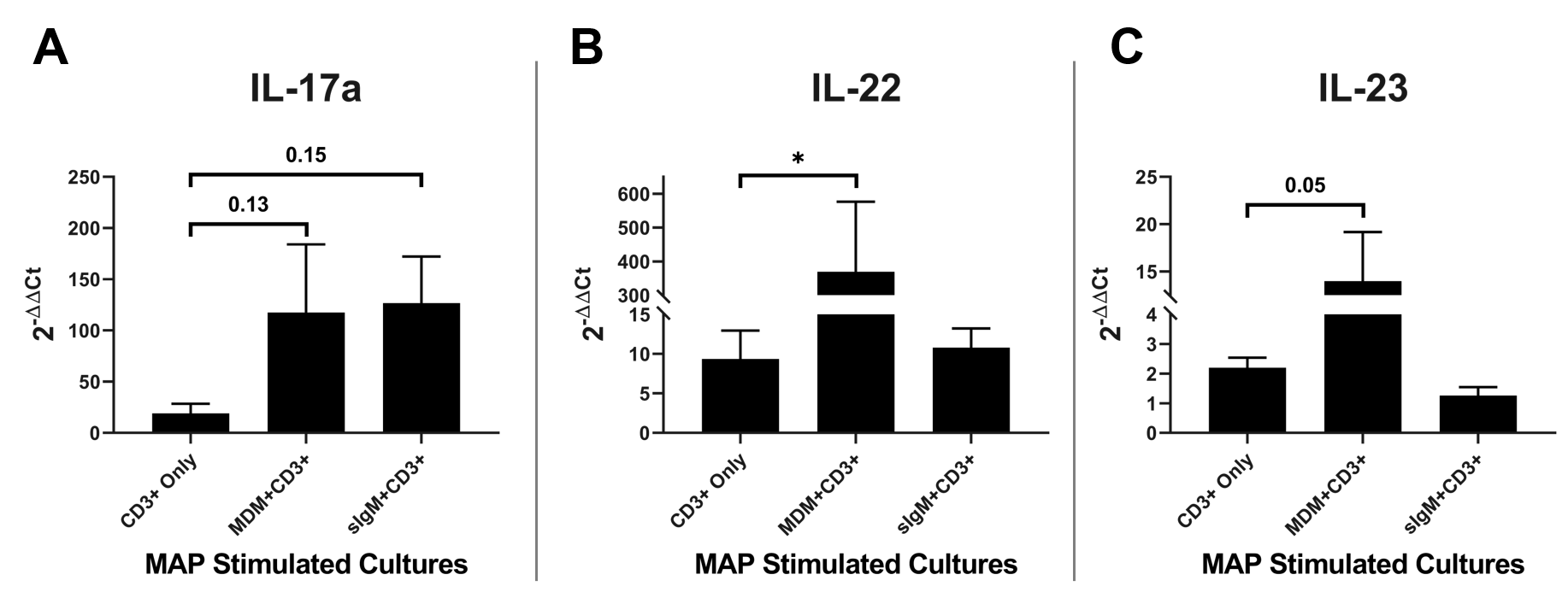

Supplement: Figure S3 — Relative abundance of IL-17A, IL-22, and IL23 mRNA of CD3+ cells, MDM/CD3+, and sIgM+/CD3+ cultures stimulated with MAP. CD3+ T cell cultures with and without APCs were stimulated with MAP for 18 h. Subsequent RNA extraction and qPCR results are shown. (A) APC containing cultures demonstrated the most upregulation of IL-17A (n = 7–8/group). (B) MDM containing cultures demonstrated the most upregulation of (B) IL-22 (n = 7–8/group) and (C) IL-23 (n = 6–8/group). Analysis by Kruskal–Wallis and Dunn's multiple comparison tests. *p < 0.05. **p < 0.01. ***p < 0.001. [file Image_3.TIF]

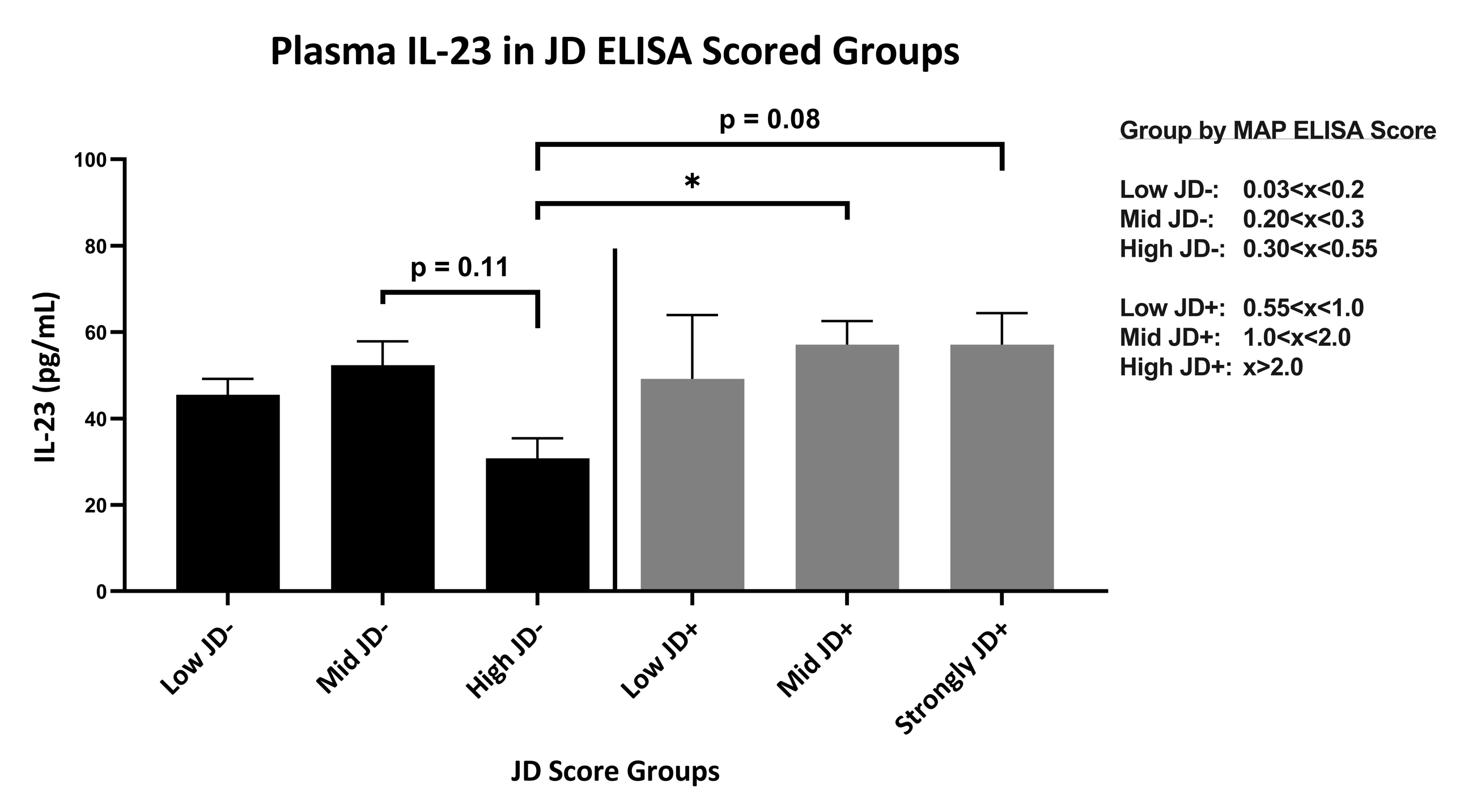

Supplement: Figure S4 — Plasma IL-23 levels of cows based on IDEXX Johne's ELISA score. IL-23 concentrations (pg/mL) circulating in the plasma from the periphery of by ELISA. Low JD– (x <0.2; n = 29). Mid JD– (0.2 < x0.3; n = 9). High JD– (0.3 < x <0.55; n = 8). Low JD+ (0.55 < x <1.0; n = 6). Mid JD+ (1.0 < x <2.0; n = 9). High JD+ (x > 2.0; n = 15). Brown-Forsythe ANOVA test and Dunnett's T3 multiple comparisons test were used in the observation of score groups. *p < 0.05. Error bars = SEM. Cow n is based on available stocked plasma samples. [file Image_4.TIF]
